# Correction of Multispectral Singlet Oxygen Luminescent Dosimetry (MSOLD) for Tissue Optical Properties in Photofrin-Mediated Photodynamic Therapy

**DOI:** 10.3390/antiox13121458

**Published:** 2024-11-28

**Authors:** Weibing Yang, Madelyn Johnson, Baozhu Lu, Dennis Sourvanos, Hongjing Sun, Andreea Dimofte, Vikas Vikas, Theresa M. Busch, Robert H. Hadfield, Brian C. Wilson, Timothy C. Zhu

**Affiliations:** 1 Department of Radiation Oncology, University of Pennsylvania, Philadelphia, PA 19104, USA; madelyn.johnson@pennmedicine.upenn.edu (M.J.); baozhu.lu@pennmedicine.upenn.edu (B.L.); dsourvan@dental.upenn.edu (D.S.); hongjing.sun@pennmedicine.upenn.edu (H.S.); andreea.dimofte@pennmedicine.upenn.edu (A.D.); theresa.busch@pennmedicine.upenn.edu (T.M.B.); 2 Department of Periodontics, School of Dental Medicine, University of Pennsylvania, Philadelphia, PA 19104, USA; 3 Center for Innovation and Precision Dentistry (CiPD), School of Dental Medicine, School of Engineering, University of Pennsylvania, Philadelphia, PA 19104, USA; 4 James Watt School of Engineering, University of Glasgow, Glasgow G12 8QQ, UK; vikas.vikas.2@glasgow.ac.uk (V.V.); robert.hadfield@glasgow.ac.uk (R.H.H.); 5 Department of Medical Biophysics, University Health Network, University of Toronto, Toronto, ON M5G 2C4, Canada; brian.wilson@uhn.ca

**Keywords:** photodynamic therapy (PDT), singlet oxygen luminescence, tissue optical properties

## Abstract

The direct detection of singlet-state oxygen (^1^O_2_) constitutes the holy grail dosimetric method for type-II photodynamic therapy (PDT), a goal that can be quantified using multispectral singlet oxygen near-infrared luminescence dosimetry (MSOLD). The optical properties of tissues, specifically their scattering and absorption coefficients, play a crucial role in determining how the treatment and luminescence light are attenuated. Variations in these properties can significantly impact the spatial distribution of the treatment light and hence the generation of singlet oxygen and the detection of singlet oxygen luminescence signals. In this study, we investigated the impact of varying optical properties on the detection of ^1^O_2_ luminescence signals during Photofrin-mediated PDT in tissue-mimicking phantoms. For comparison, we also conducted Monte Carlo (MC) simulations under the same conditions. The experimental and simulations are substantially equivalent. This study advances the understanding of MSOLD during PDT.

## 1. Introduction

Multispectral singlet oxygen (^1^O_2_) luminescence dosimetry (MSOLD) is under development for the direct detection of singlet-state oxygen near-infrared (~1270 nm) luminescence for photodynamic therapy (PDT) dosimetry [1,2,3], which is considered the holy grail dosimetric method for type-II PDT. The weak nature of the singlet oxygen (^1^O_2_) signal arises from its extremely short lifetime in biological media due to its high reactivity with biomolecules [4,5,6,7,8]. Several techniques have been developed to detect SO luminescence, including time- and wavelength-resolved spectroscopy [9,10]. Despite significant technological advances, applying these techniques in clinical settings remains a considerable challenge. Nevertheless, several research groups have successfully achieved the direct detection of ^1^O_2_ luminescence, both in vitro and in vivo, using InGaAs photodetectors. Moreover, it has been demonstrated that the ^1^O_2_ signal can be detected in clinical environments using an optical fiber probe combined with a continuous wave (CW) laser [1,2,3]. While overcoming the challenges of signal detection is crucial, it is equally important to consider the effects of tissue’s optical properties to ensure accurate and absolute PDT dosimetry using the ^1^O_2_ luminescence signal, since they significantly influence the propagation and intensity of the detected signal [10,11].

The spatial heterogeneities of optical properties for different tissue sites and variations in blood content and pigmentation are common within individual organs or tumors in the same patient and across different patients [12,13], and the distribution of treatment laser light within target tumor tissue and ^1^O_2_ detection are affected by variations in these properties [14]. This may be misinterpreted as the non-uniformity of dose deposition. Moreover, variations in optical properties lead to significant differences in the volume treated by the laser during PDT [15].

Photofrin (Pinnacle Biologics Inc., Bannockburn, IL, USA) is the first clinically approved photosensitizer and one of the most widely studied and utilized agents in photodynamic therapy (PDT) [16]. Derived from hematoporphyrin derivative (HpD), Photofrin is a mixture of oligomers formed by the ether and ester linkages of porphyrin units [17]. Upon activation by light at a wavelength of approximately 630 nm, Photofrin generates reactive oxygen species, ^1^O_2_, which induces cytotoxic effects on targeted cancer cells. Photofrin’s ability to localize in tumors is due to its preferential uptake compared to normal tissues [18].

In this study, we used both simulations and experimental approaches to evaluate the effect of tissue’s optical properties on Photofrin-PDT and ^1^O_2_ luminescence detection through optic fibers. The wavelength of the treatment laser was 632 nm and ^1^O_2_ luminescence was detected at 1270 nm. The tissue optical properties at 632 nm within the clinically relevant range were as follows: the absorption coefficient ($\mu_{a}$) = 0.1–1.0 cm^−1^ and reduced scattering coefficient ($\mu_{s}^{\prime }$) = 5–40 cm^−1^ [13]. The additional $\mu_{a}$ due to 100 mg/kg of Photofrin was measured as 0.504 cm^−1^. $\mu_{s}^{\prime }$ at wavelengths (λ) of 632 nm and 1270 nm and was approximated by the Mie scattering theory, using *μ_s_*′(λ) = Aλ^−b^ [19]. We simulated the spatial distribution of the treatment laser and the detection of the ^1^O_2_ signal using a virtual optical fiber with the same core diameter and numerical aperture (NA) as the experimental fiber. Additionally, we compared the ^1^O_2_ signal from the Monte Carlo simulation with the experimentally detected signal from Photofrin in phantoms.

## 2. Materials and Methods

### 2.1. Optical Properties Measurement for Liquid Phantom

Black India ink (Higgins Inc., Leeds, MA, USA) and Nutralipid (20%, B. Braun Medical Inc, Bethlehem, PA, USA) were used, respectively, to mimic the $\mu_{a}$ and $\mu_{s}^{\prime }$ of the tissue in liquid phantoms. The $\mu_{a}$ of the ink in methanol was measured as a function of concentration in a 1 cm cuvette using both a UV-Vis spectrometer (FLMS01765, Ocean Optics, Orlando, FL, USA) and an InGaAs spectrometer (AvaSpec-NIR256/512-1.7-EVO, Avantes, Lafayette, CO, USA). $\mu_{s}^{\prime }$ was measured as a function of Nutralipid concentration using a dual-catheter technique [20], and the Mie theory was applied to measurements of light intensity versus depth using an isotropic point detector and an isotropic point laser source.

### 2.2. Experimental Set-Up for Singlet Oxygen Detection

We conducted a series of experiments to detect the ^1^O_2_ signal from Photofrin in methanol under 632 nm laser excitation using an Avantes spectrometer with a 200 µm slit width, as illustrated in Figure 1a. This closely replicates the configuration used in previous mouse studies, where a laser with a 1 cm² spot was directed onto subcutaneous tumor grafts and a fiber detector was positioned at the side to detect the ^1^O_2_ signal [21].

To investigate how the tissue’s optical properties modulated the ^1^O_2_ spectrum, we prepared a series phantoms with $\mu_{a}$ = 0.6, 0.8, 1.0, 1.2 or 1.5 cm^−1^ (ink + 0.6 cm^−1^ from 100 m/kg Photofrin) and $\mu_{s}\prime$ = 5, 10 15, 20 or 40 cm^−1^ at 632 nm from the Nutralipid. A 632 nm CW laser (A-1030, Biolitec, Vienna, Austria) excited the photosensitizer through an optical fiber with a micro-lens tip (Pioneer Optics Co., East Granby, CT, USA) to produce a uniform 1 cm^2^ spot on the sample. A 1.5 mm core optic fiber was connected to the spectrometer to collect the ^1^O_2_ luminescence. Given that the penetration depth of the signal in the 1200 to 1600 nm range was approximately 2–3 mm [22,23], the detection volume was confined to a small, superficial layer. This localized detection highlights the advantage of using an optical fiber for signal acquisition, particularly when transitioning this technology to clinical applications where significant heterogeneity in tissue’s optical properties exists across different organs and anatomical sites.

The spectral range of 1200–1600 nm adequately covered the 1270 nm ^1^O_2_ peak. Singular value decomposition (SVD) implemented in Matlab (R2022b, The Mathworks, Matick, MA, USA) was used to fit the ^1^O_2_ spectrum using a basis function for the ^1^O_2_ peak, the background from the laser, and near-infrared fluorescence from Photofrin. The latter was obtained by quenching the ^1^O_2_ with sodium azide (NaN_3_), while the ^1^O_2_ basis spectrum was obtained by subtracting the quenched spectrum from the unquenched spectrum. The laser background was directly measured from the source.

### 2.3. Monte Carlo Modeling of Singlet Oxygen Signal

The Monte Carlo model was implemented using MCmatlab (v4.3.1) [24], which models the propagation of light in a 3D voxel space. The simulation set-up is illustrated in Figure 1b, with the dimensions matching those of the cuvette used to hold the liquid phantom (2 cm × 2 cm × 1 cm). A total of 10^7^ simulated excitation photons were launched perpendicular to the cuvette surface. The index of refraction was set at 1.4 and the anisotropy parameter (*g*) at 0.9. The reduced scattering coefficient in the Monte Carlo simulation matches the measured experimental values. An optic fiber with a diameter of 1.5 mm and a numerical aperture (NA) of 0.5 was positioned at the edge of the laser beam at a ~20° angle to the direction of the beam. The fiber collected both 632 nm and 1270 nm light. The singlet oxygen quantum yield was assumed to be 0.5, serving as a scaling factor for the generated ^1^O_2_. This assumption did not affect the overall trend of the ^1^O_2_ signal as a function of $\mu_{a}$ and $\mu_{s}^{\prime }$, provided it remained consistent throughout the simulation.

Ten million 632 nm photons were launched in each Monte Carlo simulation. The spatial distribution of the 632 nm light within the phantom was simulated from the given optical properties. The generation of the 1270 nm luminescence was then simulated from the 632 nm light distribution, and these photons were simulated with corresponding tissue absorption and scattering coefficients at this wavelength. A virtual optical fiber, matching the physical conditions of the experimental fiber, was used to collect the 1270 nm photons exiting the phantom.

## 3. Results and Discussion

Figure 2a illustrates the extinction spectrum of Photofrin from 600 to 800 nm, displaying the peak around 632 nm that is usually used in PDT treatments. Figure 2b presents the Photofrin extinction coefficient from 1200 to 1350 nm.

Figure 3a,c show the absorption coefficient of the black ink for various concentrations in the range of 600–1000 nm and 1200–1350 nm. The extinction coefficient at 632 nm and 1270 nm for 100 mg/kg Photofrin were 0.504 cm^−1^ and 0.06 cm^−1^, respectively. Focusing on the excitation wavelength of 632 nm and the emission wavelength of 1270 nm, the absorption coefficient was calculated and compared with the ink concentration as shown in Figure 3b,d. This comparison presented a linear relationship between the ink concentration and absorption coefficient. This allowed the manipulation of the absorption coefficient in the liquid phantom by adjusting the ink concentration in methanol.

Figure 4a presents the light fluence rate per unit source strength as a function of distance along the catheter, measured using an isotropic detector and a 661 nm laser source positioned in two parallel catheters. The measured spectrum was fitted using a MATLAB program based on the Mie theory, allowing the extraction of the scattering coefficients. Four phantoms were prepared with a fixed ink concentration, while the Nutralipid concentration varied from 0.3 to 0.6%. A linear relationship is seen between the measured scattering coefficient and the Nutralipid concentration in Figure 4b. The non-zero intercept in the fit likely stems from minor experimental imperfections or limitations.

Figure 5 presents the measured ^1^O_2_ luminescence spectrum of Photofrin in a turbid medium with $\mu_{a}=1.0\;{cm}^{-1}$ and $\mu_{s}^{\prime }=15\;{cm}^{-1}$. Using singular value decomposition (SVD) fitting, the singlet oxygen signal, Photofrin luminescence and laser background were separated into distinct individual curves, as illustrated in Figure 5.

Figure 6a shows spectra and the extracted ^1^O_2_ luminescence peak with a varying absorption and fixed scattering, showing an inverse dependence, the corresponding ^1^O_2_ components are displayed below the spectra. Figure 6b shows the spectra and corresponding ^1^O_2_ signal for varying $\mu_{s}^{\prime }$ with $\mu_{a}$ fixed at 1.1 cm^−1^. The ^1^O_2_ signal increases non-linearly with $\mu_{s}^{\prime }$ in the range 5–20 cm^−1^ and saturates above this. The normalized intensity of the extracted ^1^O_2_ signal and the corresponding Monte Carlo simulation results demonstrates very good agreement as shown in Figure 7.

In order to compare the experimental data and MC simulations for all 25 combinations of $\mu_{a}$ and $\mu_{s}^{\prime }$, we normalized the results to the reference point where $\mu_{a}$ = 1.0 cm^−1^ and $\mu_{s}^{\prime }$ = 5 cm^−1^, as shown in Figure 7. The excellent agreement indicates that the MC model can be used to simulate the relative ^1^O_2_ signal for any other conditions.

Based on the Monte Carlo simulations, we calculated a correction factor relative to $\mu_{a}$ = 1.0 cm^−1^ and $\mu_{s}^{\prime }$ = 15 cm^−1^ according to the following equation:

$${CF}_{\mu_{a},\mu_{s}^{\prime }}=\frac{{SO}_{ref}}{{SO}_{\mu_{a},\mu_{s}^{\prime }}}$$

The results are summarized in Table 1; we can see the correction factor can be as large as 1.97 and as small as 0.69. This large variation of the ^1^O_2_ signal due to the tissue’s optical properties indicates the necessity of using this correction factor.

## 4. Conclusions

Photodynamic therapy (PDT) is a minimally invasive medical treatment that combines a photosensitizing agent, laser light and oxygen to generate cytotoxic reactive oxygen species (ROS), predominantly singlet oxygen (^1^O_2_). However, antioxidants present in tissues can neutralize ROS by scavenging these reactive species, thereby reducing PDT’s effectiveness [26]. High antioxidant levels in tissue can diminish PDT’s efficacy by lowering the ^1^O_2_ concentration, which impacts overall therapeutic outcomes. This highlights the importance of directly detecting near-infrared ^1^O_2_ luminescence—a gold standard for accurate PDT dosimetry—since it directly measures the concentration of the primary cytotoxic agent for Type II photosensitizers.

Variations in tissue’s optical properties, such as blood oxygenation levels, antioxidant concentration and pigmentation, can markedly impact the detected signal. To investigate this, we used an Avantes spectrometer to measure the ^1^O_2_ luminescence from a liquid tissue-simulating phantom containing Photofrin in methanol, while systematically varying the scattering and absorption coefficients. This study, utilizing liquid phantoms with uniform optical properties, provides a critical foundation for exploring real tissues with significant heterogeneities across organs and sites. Further studies using mouse models are essential to validate our results and bridge the gap between phantom experiments and clinical applications.

Employing singular value decomposition (SVD), we successfully extracted the ^1^O_2_ signal from the background and Photofrin luminescence in the spectrum. Consistency was observed between the experimental results and Monte Carlo simulations for the normalized ^1^O_2_ luminescence. Correction factors were calculated as a function of absorption and scattering, normalized to a specific combination of absorption ($\mu_{a}$ = 1.0 cm^−1^) and scattering ($\mu_{s}^{\prime }$ = 15 cm^−1^). The detected singlet oxygen signal exhibited an approximately linear decrease with the tissue absorption coefficient, while it increased with the scattering up 20 cm^−1^, after which it began to saturate. While these results apply to a sample and irradiation/detection geometries, they demonstrate the strong dependence of the detected singlet oxygen luminescence signal on the tissue’s optical properties. Hence, independently measuring the absorption and scattering coefficients of target tissue at the treatment wavelength and around 1270 nm, for example, by diffuse reflectance spectroscopy [27], should significantly improve the accuracy of singlet oxygen luminescence dosimetry for PDT.

In addition, the close agreement between the Monte Carlo simulations and the experimental data for this particular set-up indicates that the same approach may be used with some confidence to determine the corresponding correction factors for other target tissue, irradiation and detector geometries. The concordance between the experiment and simulations also further validates the use of MSOLD as a singlet oxygen luminescence dosimetry technique.

## Figures and Tables

**Figure 1:**
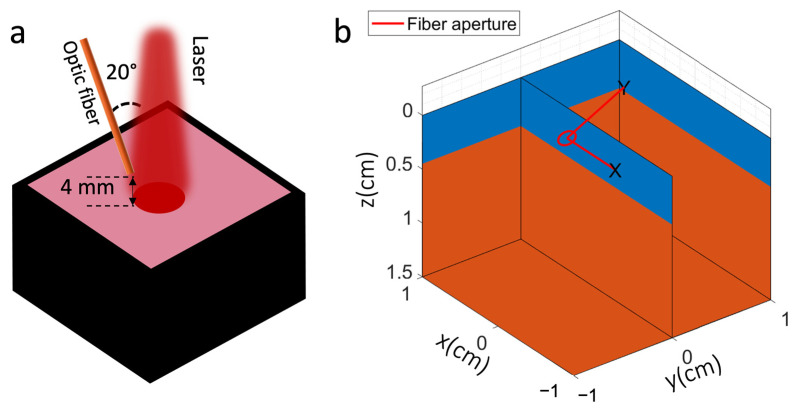
(**a**). Schematic illustration of the experimental set-up in which the laser strikes the cuvette along the z-axis and the detection fibers with NA = 0.5 are placed at 20 ± 2° to the laser beam with the fiber tip 4 ± 0.5 mm above the surface, the pink color represents the Photofrin solution, while the black color represents the black phantom. (**b**) Schematic illustration of the Monte Carlo simulation configuration, mirroring the experimental set-up, the red region represents the liquid phantom, while the blue region represents air.

**Figure 2:**
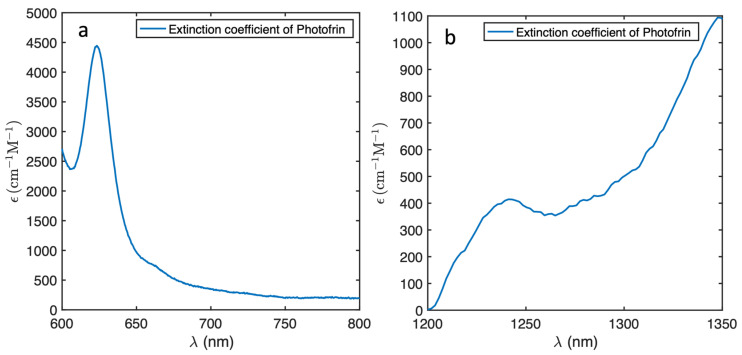
The extinction coefficient spectrum of Photofrin around the excitation (**a**) and ^1^O_2_ luminescence emission (**b**) wavelengths. The former is consistent with the values in the literature [25].

**Figure 3:**
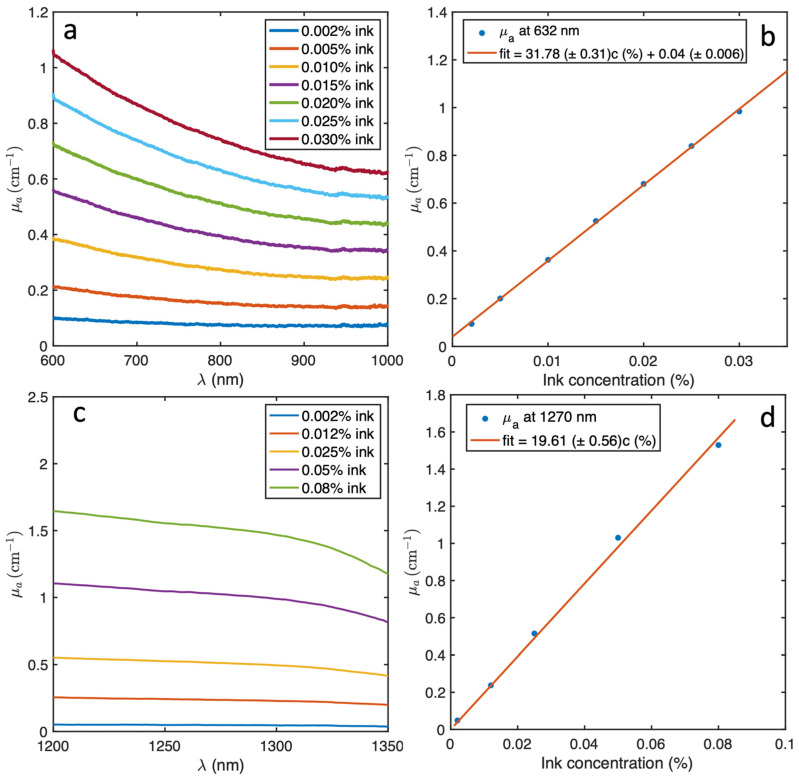
(**a**) The absorption spectrum of the black ink from 600 nm to 1000 nm. (**b**) The calculated absorption coefficient at 632 nm as a function of the concentration, together with a linear fit with the measurements. (**c**,**d**) Corresponding measurements from 1200 to 1350 nm and at 1270 nm.

**Figure 4:**
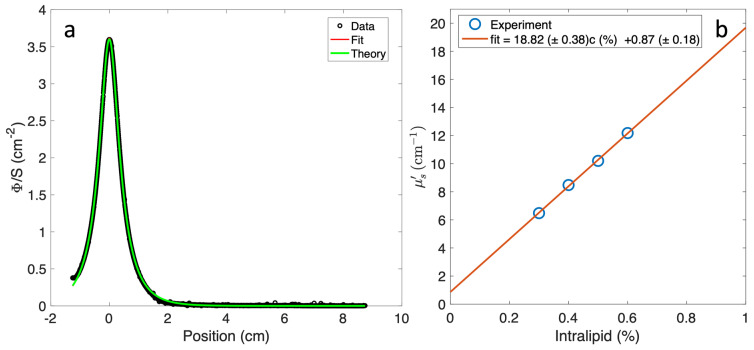
(**a**) An example of the measured light fluence rate per unit source strength (Φ/S) at 661 nm versus the distance along the catheter from a point source: fitted $\mu_{s}^{\prime }=8.5\;{cm}^{-1}$. (**b**) The reduced scattering coefficient from (**a**) at 661 nm for Intralipid as a function of the concentration and the corresponding linear fit.

**Figure 5:**
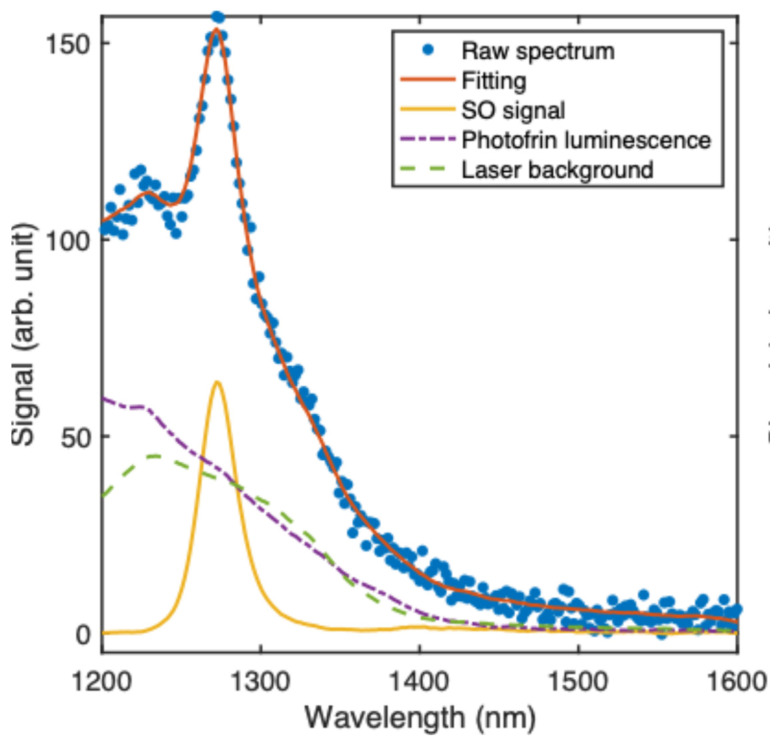
Singlet oxygen luminescence spectrum of Photofrin, measured in a turbid phantom with $\mu_{a}=1.0\;{cm}^{-1}$ and $\mu_{s}^{\prime }=15\;{cm}^{-1}$, together with the calculated component spectra.

**Figure 6:**
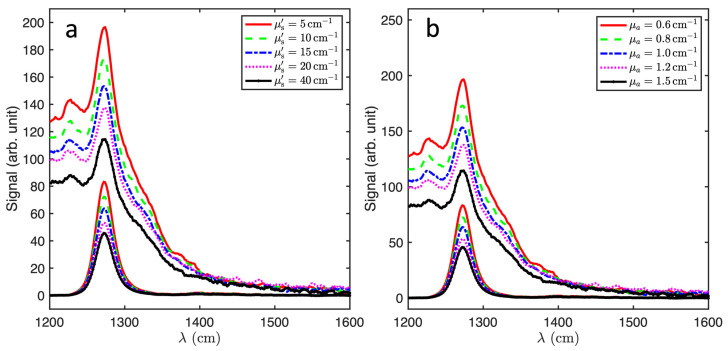
Measured singlet oxygen luminescence spectra with 10-point smoothing and the corresponding spectra obtained by SVD fitting. (**a**) $\mu_{a}$ ranging from 0.6 to 1.5 cm^−1^ at 632 nm (ink + Photofrin contributions) with $\mu_{s}^{\prime }$ fixed at 15 cm^−1^. (**b**) Corresponding spectra with $\mu_{s}^{\prime }$ = 5–40 cm^−1^ and $\mu_{a}$ = 0.5 cm^−1^ at 632 nm.

**Figure 7:**
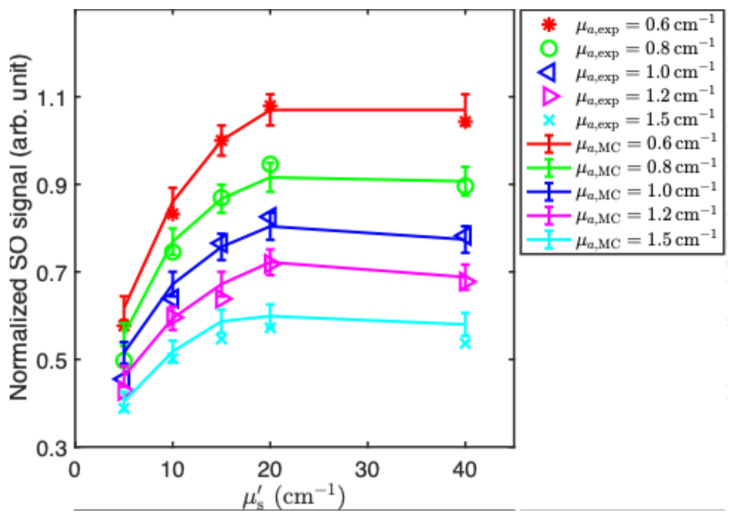
Dependence of the normalized ^1^O_2_ signal on the scattering coefficient at 632 nm, comparing the experimental data (points) and the Monte Carlo simulations (solid lines), normalized to the values of $\mu_{a}$ = 0.8 cm^−1^ and $\mu_{s}^{\prime }$ = 15 cm^−1^.

**Table 1:** Correction factor for tissue’s optical properties for 100 mg/kg Photofrin ($\mu_{a}$ = 0.6 cm^−1^) as a function of $\mu_{a}$ (ink + Photofrin) and $\mu_{s}^{\prime }$ at 632 nm, normalized to Monte Carlo simulation at $\mu_{a}$ = 1.0 cm^−1^ and $\mu_{s}^{\prime }$ = 15 cm^−1^.

$\mu_{s}^{\prime }$ (cm^−1^)	$\mu_{a}$ (cm^−1^)				
	0.6	0.8	1.0	1.2	1.5
5	1.22	1.36	1.47	1.64	1.87
10	0.88	0.99	1.13	1.28	1.46
15	0.76	0.87	1.0	1.13	1.29
20	0.71	0.83	0.94	1.05	1.26
40	0.71	0.84	0.98	1.10	1.31

## Data Availability

The data will be made available upon request.
